# Global health security and the International Health Regulations

**DOI:** 10.1186/1471-2458-10-S1-S2

**Published:** 2010-12-03

**Authors:** Jon Kim Andrus, Ximena Aguilera, Otavio Oliva, Sylvain Aldighieri

**Affiliations:** 1 Pan American Health Organization, 525 23rd Street, NW, Washington, DC, 20016, USA; 2 Center of Epidemiology and Public Health Policy, Universidad del Desarrollo Clínica Alemana de Santiago, Chile (Formerly Staff at the Pan American Health Organization

## Abstract

Global nuclear proliferation, bioterrorism, and emerging infections have challenged national capacities to achieve and maintain global security. Over the last century, emerging infectious disease threats resulted in the development of the preliminary versions of the International Health Regulations (IHR) of the World Health Organization (WHO). The current HR(2005) contain major differences compared to earlier versions, including: substantial shifts from containment at the border to containment at the source of the event; shifts from a rather small disease list (smallpox, plague, cholera, and yellow fever) required to be reported, to all public health threats; and shifts from preset measures to tailored responses with more flexibility to deal with the local situations on the ground. The new IHR(2005) call for accountability. They also call for strengthened national capacity for surveillance and control; prevention, alert, and response to international public health emergencies beyond the traditional short list of required reporting; global partnership and collaboration; and human rights, obligations, accountability, and procedures of monitoring. Under these evolved regulations, as well as other measures, such as the Revolving Fund for vaccine procurement of the Pan American Health Organization (PAHO), global health security could be maintained in the response to urban yellow fever in Paraguay in 2008 and the influenza (H1N1) pandemic of 2009-2010.

## Introduction

Traditional infectious disease threats like smallpox, plague, yellow fever, and cholera prevented nations from ensuring the stability and well-being of their populations for centuries. In some countries, such diseases contributed to border conflicts and other disputes. Accordingly, these diseases have long been considered in the development of international treaties. Early attempts to control them led to the actual development of public health programs in most countries. More recently, the global nuclear proliferation, bioterrorism, and emerging infections, such as new strains of influenza, human immuno-deficiency virus (HIV), and multi-drug resistant infections have further challenged national capacities to achieve and maintain global security [1].

The purpose of this article will be to provide an overview of the global health security in the context of the development of the International Health Regulations (IHR). Topics to be covered include: milestones leading to the current version of the IHR, the critical elements of the functions of the IHR, and recent examples of IHR implementation to control the urban yellow fever outbreak in Paraguay in 2008 and the A/H1N1 influenza pandemic of 2009-2010.

## IHR development milestones

For centuries, diseases such as “black death” and smallpox killed millions of people worldwide. It was not until the 1700s that the first effective public health intervention was implemented widely. Quarantine of persons with contagious disease in some well-managed situations had a dramatic effect on reducing disease transmission. Other grossly inadequate interventions, such as phlebotomy of patients or toxic spraying of neighborhoods, continued to be used in the face of scientific and public ignorance.

In 1950, smallpox, cholera, plague, and yellow fever continued to kill thousands of people worldwide. Most deaths occurred in the poorest countries. Wealthier countries fretted over the risk of importations and the risk of outbreaks in their national populations. These concerns led to the first version of the IHR, drafted in 1951, which was subsequently revised and strengthened in 1969. However, the new IHR did little to curb the persistence of international threats. For example, the last three polio outbreaks in the United States occurred in the 1970s, ultimately as a result of importations of wild poliovirus from the Indian sub-continent. By the next decade, the acquired immunodeficiency syndrome (AIDS) pandemic had emerged to impose enormous challenges to global health security.

By 1990, in countries of Latin America and the Caribbean the polio eradication initiative was well underway to achieve the eradication target [2]. However, the program was severely threatened by the emergence of a cholera epidemic in Peru in early 1991. The epidemic spread rapidly and affected virtually every country in the Western Hemisphere. Interestingly, the island of Hispaniola was not affected. To control the epidemic, countries such as Colombia used polio vaccination campaigns that included messages on safe water, hygiene, and sanitation, in order to prevent the spread of cholera. The opportunity to interrupt cholera transmission was more fortuitously linked to the existing polio eradication program than to any planned procedures or intended influence of the IHR.

In 1994, Surat, India, was reported to have plague by the Ministry of Health of India [3]. A global alarm was sounded largely through the reporting done by the press. Despite debates about definitive occurrence of laboratory confirmed cases in the initial phases of the outbreak and the appropriateness of certain interventions, airports were closed; travel was disrupted both nationally and globally; embassies were closed; and some embassies even sent their staff home to their respective countries. All this happened with a tremendous economic cost to the peoples and Government of India. Clearly, the IHR had little effect on preventing the rather rash actions taken.

The next year Ebola erupted in Kitwit, Africa [4]. The WHO spearheaded its first global and coordinated response to such an emerging threat. Recognizing the inadequacies of the Surat experience, in May 1995 the WHO declared that the 1969 version of the IHR was obsolete and requested its revision. By the late 1990s, a new meningococcal bacteria strain (w135) emerged in West Africa. Building on the lessons learned from the first Ebola response, the WHO organized the Global Outbreak Alert and Response Network (GOARN). This global mechanism was immediately challenged to address major outbreaks like the re-emergence of the Ebola and Marburg virus threats in Africa at the beginning of the 2000-2010 decade. Because most developing countries had little capacity to respond to threats nationally, the WHO at that time also established the National Epidemic Preparedness and Response team in their headquarters.

In 2003, the world was shocked again with the first global pandemic of severe acute respiratory syndrome (SARS) [5]. The case-fatality ratio of SARS approached 10%, and 20% of the cases were among health care workers. Although difficult to quantify, the WHO estimated that the global cost of this pandemic probably ranged from USD 30-50 billion. Concerns over national capacity and the accountability of nations to report global threats led to the 2005 version of the IHR, known as IHR(2005), that is currently in use today.

## Critical elements and functions of the IHR(2005)

The purpose of IHR(2005) is “to prevent, to protect against, control, and provide a public health response to the international spread of disease in ways that are commensurate with and restricted to public health risks, and which avoid unnecessary interference with international traffic and trade” (Article 2) [6]. As such, major differences between the current version and previous versions include the following:

• Shift from containment at the border to containment at the source of the event;

• Shift from a rather small disease list (smallpox, plague, cholera, and yellow fever) required to be reported, to all public health risks, including chemical and radio nuclear threats; and

• Shift from preset measures to tailored responses with more flexibility to deal with the local situations on the ground and the advice of the emergency committee.

The new IHR(2005) call for accountability. Account ability in reporting critical events is inextricably linked to the national or local capacity to identify the etiology. Confusion over the diagnosis will delay reporting that will ultimately affect global health security, no matter how responsible and committed a particular nation is. Therefore, the IHR(2005) also call for strengthened national capacity for surveillance and control; prevention, alert, and response to international public health emer-gencies beyond the traditional short list of required reporting; global partnership and collaboration; and rights, obligations, accountability, and procedures of monitoring [6].

The new IHR(2005) include a broad scope of work. Case definitions of diseases, public health emergency of international concern (PHEIC), and public health risks are intended to be more unambiguous with analytic tools to evaluate every potential public health risk of international concern. Additionally, biological, chemical, and radio nuclear etiologies are included and implicit. Collaboration with other mandated organizations is absolutely essential. At the country level, the IHR(2005) are supported by the designation of national IHR focal points. Each focal point is mandated to promote efficient and accurate communications in emergencies; coordinate with existing mechanisms such as the WHO Country Representative, International Food Safety Authorities Network (INFOSAN), and Chem Alert; and prioritize national engagement and ownership. A key priority is to strengthen national capacity for surveillance and response. What happens in countries is considered most important and a key element of the WHO strategy for global health security [6].

Mechanisms for advice and oversight of national capacity development are:

• National roster of experts that can be called upon immediately to deal with any crisis as it occurs;

• Emergency committees to manage the response;

• Review committees to monitor progress and define lessons learned from the way each event was managed so that corrective action can be taken for future events; and

• Global support through policy development at the World Health Assembly (WHA) and regional committees of the WHO.

## Examples of recent challenges to the IHR(2005) Yellow fever

For the first time in more than 45 years in the Western Hemisphere, Paraguay reported an urban yellow fever outbreak in the peri-urban area its national capital, Asuncion, in 2008 [7]. Ten people died a few short weeks later. Annually, neighboring Brazil reports episodic jungle yellow fever, which is different from the urban pattern. Jungle yellow fever occurs in tropical rainforest areas where wild mosquitoes, *Haemagogus*, can infect monkeys with yellow fever virus. Monkeys serve as the reservoir for other wild *Haemagogus* mosquitoes to become infected with the virus. Infected mosquitoes can transmit the yellow fever virus to humans who enter the rainforest. Human victims of jungle yellow fever infection are typically young males who work or hunt in the rainforest. Urban yellow fever occurs when domestic mosquitoes, *Aedes aegypti*, transmit the virus to humans [8]. Urban transmission is not dependent upon the monkey reservoir, so outbreaks can be explosive and cause substantial mortality quickly. In cases of urban transmission, the case-fatality ratio approaches 40% [9].

In response to the urban yellow fever outbreak in Paraguay, the IHR(2005) were activated. Once notified, the WHO in Geneva, the WHO Regional Office for the Americas (known as the Pan American Health Organization, or PAHO), and the Paraguayan Ministry of Health initiated emergency discussions to mobilize yellow fever vaccine from the global stockpile. In addition, PAHO deployed a team to provide technical cooperation to enhance surveillance, outbreak investigation, vector control, risk communication, and logistic operations. PAHO also helped mobilize laboratory expertise and critical reagents to conduct the investigative testing. Rapid coordination with the Ministry of Health was essential to the response.

The global response to the urban yellow fever outbreak in Paraguay resulted in the rapid mobilization of yellow fever vaccines. The PAHO Revolving Fund, created for the purchase and management of the supply of vaccines, played a critical role in mobilizing the vaccine from nations around the southern hemisphere. The global stockpile of yellow fever vaccine had insufficient number of vaccine doses to respond effectively to the crisis in Paraguay. As a result, efforts of PAHO, in coordination with other PAHO Member States, led to vaccines being mobilized from national stockpiles of neighboring countries in the region for use in Paraguay. Paraguay was able to implement a timely and rapid vaccination response. Before the arrival of vaccines, civil disturbance was mounting in the face of public panic. These regional and global efforts prevented many more deaths, as well as the potentially disastrous consequences of uncontrolled civil disturbance. The political and technical collaboration led to improved global health because the outbreak was stopped, and also served to enhance political relations in the region. There were other global health consequences. Had the outbreak continued, Africa would have faced a serious threat due to insufficient global vaccine supply to respond to potential outbreaks. The IHR(2005) mechanism put the world on alert and contributed to the resulting successful global response, but other national and regional capacities, such as the PAHO Revolving Fund, contributed substantially as well [7,10].

### A/H1N1 infuenza

On April 18, 2009, the national IHR focal point of the United States notified the WHO through the IHR(2005) mechanism of the detection of a new strain of A/H1N1 influenza virus in two boys, one of whom had a travel history to Mexico [11]. Soon after, this alert led to the detection of the same virus in Mexico, where an outbreak of severe respiratory illness had been evolving for some weeks. The first IHR(2005) Emergency Committee meeting was convened on April 25, a Saturday. As a result, the WHO declared a public health emergency of international concern. On June 11, the Director General of the WHO, Dr. Margaret Chan, declared influenza pandemic phase 6. By August 2010, over a year later, she announced that the pandemic had subsided.

During its course, the A/H1N1 influenza pandemic killed over 18,000 people and infected thousands of people across the globe in virtually every country on the planet. The global alert and response to this pandemic has been described above. The WHO, partners, and countries around the world activated their emergency operations centers, initiating disease detection, reporting, and extensive investigations. Laboratory reagents and supplies, technical experts, antiviral medications, and other protective equipment were deployed to countries in need. In the first month of the PHEIC declaration, PAHO supported the installation of the pandemic H1N1 (2009) viral diagnostics in all countries of the region in an unprecedented demonstration of international cooperation. Later, a global response coordinated vaccine distribution when vaccines became available. Fortunately, the outbreak was not as severe as many predicted, but even so, many pregnant women died. Mortality as a result of the pandemic, in fact, still needs to be evaluated more accurately. Many experts concluded that the response was necessary. If the pandemic had been more severe, the consequences would have been enormous. Without the previous work on preparedness, advance work on antiviral stocks, and written plans for global coordination through IHR mechanisms, great loss of life and chaos would surely have ensued. Most experts would conclude that the technical cooperation provided to the pandemic control was unprecedented and very appropriate.

## Conclusions

The global public health community has unprecedented support to respond to global pandemics and public health emergencies of international concern. Efforts to strengthen national capacity to respond are essential and must continue well into the future. The IHR(2005) focus on accountability and capacity development has proven to be essential in the response to emergencies. In the Americas, other measures like the PAHO Revolving Fund for vaccine mobilization and purchase have also been critical. Ultimately, adherence to accountable and timely reporting of PHEIC and the national capacity to respond to such crises will prevent disruptions in the global health security and loss of life.

## Abbreviations

AIDS: Acquired immunodeficiency syndrome; GOARN: Global Outbreak Alert and Response Network; HIV: Human immunodeficiency virus; IHR(2005): The current version of the International Health Regulations that countries of the world are implementing; Infuenza A(H1N1): The pandemic strain of influenza that emerged from Mexico in early 2009; INFOSAN: International Food Safety Authorities Network; PAHO: Pan American Health Organization, the WHO Regional Office for the Americas; PHEIC: Public health emergencies of international concern; SARS: Severe acute respiratory syndrome; WHA: World Health Assembly; WHO: World Health Organization.

## Competing interests

None of the authors have a conflict of interest.

## Authors’ contributions

Dr. Andrus is the primary author, and as such wrote the paper and provided the backup reference documentation. Dr. Aguilera helped develop the oral slide presentation that was subsequently used as an outline for the text of the paper and contributed to technical reviews of the first drafts of the paper. Drs. Oliva and Aldighieri provided technical input and contributed to the editing and technical reviews of the paper.
